# Prognostic Factors in Acute Methadone Toxicity: A 5-Year Study

**DOI:** 10.1155/2014/341826

**Published:** 2014-08-12

**Authors:** Abbas Aghabiklooei, Maryam Edalatparvar, Nasim Zamani, Babak Mostafazadeh

**Affiliations:** ^1^Department of Legal Medicine and Toxicology, Iran University of Medical Sciences, Tehran, Iran; ^2^Department of Clinical Toxicology, Loghman Hakim Hospital, Shahid Beheshti University of Medical Sciences, Tehran 1333431151, Iran; ^3^Department of Forensic Medicine and Toxicology, Shahid Beheshti University of Medical Sciences, Tehran, Iran

## Abstract

*Background*. Delayed or recurrent profound respiratory depression, ventricular dysrhythmias, acute lung injury, and death are the major complications of MTD overdose. We aimed to clarify the prognostic factors in MTD toxicity. *Materials and Methods*. Retrospectively, medical files of all patients poisoned by MTD and older than 12 years of age who had presented to Loghman Hakim Poison Center between 2007 and 2012 were evaluated. The data was compared between survivors and nonsurvivors. *Results*. Twenty-eight out of 322 patients died (mortality rate = 8.7%). MTD-related death was higher in patients with acute on chronic toxicity who were on daily dose of MTD and had ingested higher doses (in comparison to those with acute toxicity due to first-time exposure; 13% versus 6%). Renal failure was the most common medical complication related to deaths due to MTD toxicity. *Conclusions*. Based on previous researches, the most common cause of MTD overdose-related deaths is respiratory impairment; however, in our study, acute renal failure with or without rhabdomyolysis was the main delayed cause of deaths in MTD-poisoned patients. Antidotal therapy, early recognition, and treatment of hemodynamic compromise and rhabdomyolysis can be life-saving in these patients.

## 1. Introduction 

Methadone (MTD), as a synthetic opioid with long elimination half-life, is the preferred drug for the treatment of opioid dependence and control of pain in Iran. This drug is available in oral liquid formulation and tablets, has analgesic effects, and can be used in the management of severe chronic pain. Patients who refer after acute overdose are generally those who obtain multiple doses of “take home” methadone from methadone maintenance therapy (MMT) clinics [1–8].

In Iran, syrup of MTD is widely prescribed for treatment of opioid addiction. It is a colorless liquid usually kept in water bottles at home and accidental acute poisoning with it is extremely common especially in children. Suicidal attempt with MTD syrup has also become so popular within the recent years [9].

Delayed or recurrent respiratory depression, ventricular dysrhythmias, acute lung injury, and death are the major complications of MTD overdose [10]. Respiratory arrest is the main cause of MTD-related deaths and usually develops 12 hours after the ingestion of the drug [4]. Additionally, torsade de pointes (TdP)—the arrhythmia induced by QT interval prolongation—may contribute to death [11, 12]. Overdose may cause renal failure as a result of rhabdomyolysis and myoglobinuria in prolonged immobilization or coma [4]. Some studies reported a 10-fold increase in MTD toxicity-related fatalities between 2000 and 2003 which could probably be because of its arbitrary use and use in pain clinics [13, 14].

It should be noted that many of MTD intoxication deaths are preventable with cautious prescription, good monitoring, and attention to the signs and symptoms of toxicity and the drug interactions [4]. It is important to have a broad knowledge about the different epidemiological aspects of MTD usage and overdose. This study aimed to describe the demographic data of MTD-overdosed patients and compare MTD-intoxicated survivors and nonsurvivors to investigate the possible prognostic factors in this toxicity.

## 2. Materials and Methods 

In this retrospective, cross-sectional study, medical data of all MTD-poisoned patients older than 12 years who had been admitted to toxicology ward of Loghman Hakim Hospital between 2007 and 2012 were evaluated. The diagnosis of MTD poisoning was based on history of its ingestion taken from the patients and/or their relatives, the presence of a minimum of two signs of opioid toxicity including central nervous system depression, respiratory depression, and miosis in physical examination, and the positive urine drug screen test for MTD (urine was checked twice, once at emergency department [ED] and the second time, 8 hours later at ward). All the available information including the demographic data, past medical history, signs and symptoms on presentation, laboratory findings, complications, and outcome was recorded. The cases with insufficient data, those with background diseases (i.e., ischemic heart disease, chronic renal failure, and advanced liver disorders), concomitant head trauma, and multidrug toxicity, and those on MTD treatment in MMT program and/or overdosed on other opioids were excluded from the study. Data analysis was performed by statistical package for social sciences (SPSS) version 16. A *P* value less than 0.05 was considered to be statistically significant. Finally, the patients were divided into two groups, those with acute toxicity after single ingestion of a dose of MTD for the first time (suicide attempt or accidental) and those with acute on chronic toxicity poisoning that were on daily doses of MTD and had ingested it more than the usual dose (suicide attempt or recreational overdose).

The data was then compared between the survivors and nonsurvivors to investigate prognostic factors. The study was performed according to the Helsinki declaration and approved by our local ethics committee.

## 3. Results 

A total of 456 MTD-poisoned patients had been admitted to toxicology ward of Loghman-Hakim Hospital between 2007 and 2012. Of them, 322 patients older than 12 years of age (mean age: 36.0 ± 15.8 years) were included. The male/female ratio was 2.97 and 241 cases (74.8%) were male. Methadone syrup had been used by 129 patients (41.4%) while others had overdosed on MTD tablets. The mean ingested dose of MTD was 85.91 ± 82.61 mg (range; 5 to 500 mg). Mean time elapsed between MTD ingestion and admission was 9.41 ± 9.87 hours (range; 1 to 72 hours).

In the first visit at ED, the most common symptom was vomiting (12.1%) and in physical examination—focused on cardinal signs of toxicity—apnea, respiratory depression (respiratory rate [RR] < 10/min), and miosis were detected in 23 (7.1%), 117 (36.3%), and 177 (60.78%) patients, respectively. Almost 32.5% of the cases were fully conscious on presentation while 59.1% were confused and sleepy, and 3.8% were in comatose status. Based on the mode of toxicity, MTD poisoning had intentionally happened (suicidal attempt, illicit use, or recreational use) in 270 patients (84.1%).

Naloxone had been administered to 126 (39.1%) patients at ED. Tracheal intubation had been performed in 79 (24.5%) patients at ED or during hospitalization due to respiratory compromise. The most common complication related to MTD poisoning was renal failure in 16 patients, in eight of whom, concomitant clinical and laboratory signs of rhabdomyolysis were present. Seven patients had rhabdomyolysis without renal failure. Thirteen of 16 patients with acute renal failure had died.

Of 322 patients, 294 survived from the poisoning and were discharged. Death was recorded in 28 cases (8.7%).  Table 1 compares the different recorded variables between survivors and nonsurvivors. The recorded lab data is summarized in Table 2.

Electrocardiographic (ECG) findings of 151 patients on admission were available and interpreted by a single cardiologist. The mean PR, ORS, and corrected QT intervals (QTc) were 150.3 ± 32.8 (range; 80 to 280) msec, 92.2 ± 21.6 (range; 40 to 150) msec, and 446.3 ± 50.7 (range; 289 to 584) msec, respectively. The comparison between ECG findings in survivors and nonsurvivors is summarized in Table 3. The complications related to MTD poisoning are shown in Table 4.

A total of 198 patients (61.5%) had intentionally or accidentally ingested MTD for the first time and 12 (6%) of them had died. Acute on chronic poisoning had happened in 123 patients (38.2%) who were users of daily doses of MTD. Of them, 92 (28.6%) were on MMT program and had ingested a higher dose of MTD to suicide or for recreational use and 31 (9.6%) had ingested it without prescription. Sixteen of these 123 patients (13%) had died. In one patient, it was not clear if the overdose was due to the first-time use or he was an abuser.

## 4. Discussion 

Methadone is a synthesized opioid with excellent oral bioavailability of 70% to 90% and a long plasma half-life (15 to 52 h) [4]. With many drug interactions, its pharmacokinetics are dependent on host factors, other consumed medications, and age [14]. Tablets or syrup of MTD are broadly prescribed for treatment of opioid dependency in Iran, and therefore, MTD-related death has increased in this country within the recent decades.

Respiratory failure is the main cause of death in these patients. Profound hypoxia due to respiratory depression or arrest can lead to acute lung injury or acute respiratory distress syndrome (ARDS), brain injury, and renal or liver impairment. Aspiration pneumonia may ensue due to decreased level of consciousness. Some MTD-intoxicated patients need endotracheal intubation and artificial ventilation because of central hypoventilation and inability to protect airways. These factors make the patients susceptible to ventilator-associated pneumonia. Dangerous TdP dysrhythmia is another uncommon cause of death in these patients.

In our study, the mortality rate was 8.7%. The mortality rate was significantly less in those who had ingested MTD for the first time (*P* = 0.04). This means that acute toxicity is more dangerous in the patients who are on MMT program. Reviewing the literature shows different but increasing rates of MTD-related fatalities in different parts of the world [10]. For instance, in Western Australia, Vermont, Ontario, and Zurich, a total of 84 (1993–1999), 76 (2001–2006), 54 (2004), and 146 (1998–2007) deaths have been reported due to MTD overdose [15–18]. Our study is one of the first reports on MTD-related deaths from the capital of Iran. The results showed that mean age of nonsurvivors was more than survivors (46.2.±21.2 versus 35.0 ± 14.9 years, resp.; *P* = 0.01). It can be concluded that MTD poisoning can contribute to more complications in older ages and surely needs more medical attention in them. The mean age of our patients was similar to a Norwegian study (36 ± 15 versus 36 ± 10 years, resp.) [3].

According to our results, there was no difference in MTD formulation between the two groups. Also, against our expectation, the mean ingested dose was lower in nonsurvivors whose possible cause is their delayed hospital presentation. On the other hand, factors including older ages, acute on chronic toxicity, severe loss of consciousness, and decreased mean arterial pressures on admission were significantly associated with poorer outcomes. It should be noted that, in our cases, respiratory rate was significantly more in nonsurvivors. Since acute lung injury, ARDS, and pneumonia [Table 4] may occur and accompany a higher respiratory rate, it can be suggested that tachypnea can be a predictor for poor prognosis in MTD poisoning.

Although no statistically significant difference was detected in the level of creatinine phosphokinase (CPK) between the survivors and nonsurvivors, CPK was less in those who survived (1691 ± 3457 versus 16058 ± 31749). Acute renal failure was a poor prognostic factor for MTD toxicity-related deaths since eight dead cases had acute renal failure due to rhabdomyolysis and acute tubular necrosis.

The frequency of endotracheal intubation, duration of hospitalization, lactate dehydrogenase (LDH), blood urea nitrogen (BUN), creatinine (Cr), aspartate transaminase (AST), and alanine transaminase (ALT) were significantly higher in the nonsurvivors, while mean platelet count was significantly lower in this group. These variables can be used as prognostic factors of MTD poisoning. Arterial pH and HCO_3_ were lower and PCO_2_ was higher in nonsurvivors. Although the differences were not significant, these findings are due to central respiratory depression and profound hypoxia in severe MTD toxicity.

Prolongation of PR (>200 msec), QRS widening (>120 msec), and prolongation of QTc interval (>450 msec) were seen in 3.3%, 7.9%, and 45% of the patients, respectively. None of these factors differed significantly between the two groups (Table 3). Previous studies had defined a QTc interval greater than 450 msec as a risk factor for cardiac dysrhythmia (such as TdP), syncope, and cardiac sudden death [10, 12, 19]. QTc interval was reported to be 472.72 ± 18.5 msec in Iranian causalities receiving MMT and prolonged in 25% of the 100 studied patients [2]. Mean dose of MTD had a significant relationship with the mean QTc interval (*P* = 0.009). There are different reports about the relationship between the mean dose of MTD and QTc interval; some show significant and others nonsignificant relationships [20, 21]. According to the literature, sudden death due to cardiac arrhythmia and especially QTc interval can occur in a wide range of doses of MTD, even in daily doses of less than 30 mg/day [19].

Higher numbers of acute renal failure, rhabdomyolysis, aspiration pneumonia, hepatic failure, sepsis, and disseminated intravascular coagulation in nonsurviving patients suggest that these patients have serious complications which can lead to death. In a study performed in London in 2004, reduced respiratory rate, aspiration, pulmonary edema, bronchopneumonia, and heart and renal problems were reported as factors causing death in MTD abusers [4].

## 5. Conclusions 

Delayed hospital presentation, severe loss of consciousness, acute toxicity in patients who were on daily dose of MTD (acute on chronic toxicity), need for early endotracheal intubation, tachypnea (as a sign of acute lung injury or aspiration pneumonitis), acute renal failure, and rhabdomyolysis can be assumed as early poor prognostic factors for MTD-related death.

## Figures and Tables

**Table 1:** Comparison of demographic data and clinical findings between survivors and nonsurvivors.

		Outcome		
		Survivors *N* = 294	Nonsurvivors *N* = 28	*P*
Demographic data	Age	35.0 ± 14.9	46.3 ± 21.2	0.01
	Sex			
	Male	73.8%	85.7%	N/S
	Female	26.2%	14.3%	
	Mode of toxicity			
	Intentional	84.3%	82.1%	N/S
	Accidental	15.7%	17.9%	
	Patient on MMT program			
	Yes	43.1%	82.4%	0.002
	No	56.9%	17.6%	

Clinical finding at ED	MTD formulation			
	Tablet	56.5%	76%	N/S
	Syrup	43.5%	24%	
	Ingested dose	87.14 ± 87.74	69.76 ± 44.11	N/S
	Duration between consumption and admission (hours)	8.94 ± 8.99	14.92 ± 16.40	N/S
	Loss of consciousness			
	Confused	45.6%	35.7%	<0.001
	Sleepy	13.6%	17.9%	
	Stuporous	3.4%	17.9%	
	Comatose	1.7%	25%	
	Pupil size in the first examination			
	Miotic	61.2%	57.1%	N/S
	Mid-sized	35.4%	39.3%	
	Dilated	3.4%	3.6%	
	Pulse rate	86.7 ± 16.5	91.3 ± 27.3	N/S
	Respiratory rate	15.98 ± 6.46	22.8 ± 12.9	0.01
	Blood pressure (mmHg)			
	Systolic	117.96 ± 19.89	105.93 ± 19.55	0.002
	Diastolic	73.60 ± 12.05	67.50 ± 8.39	0.01
	Naloxone therapy			
	Yes	39.8%	35.7%	N/S

Findings during hospitalization	Endotracheal intubation	17.7%	96.4%	<0.001
	Mean duration of hospitalization (days)	56.4 ± 79.3	181.5 ± 214.8	0.005

N/S: statistically nonsignificant (*P* > 0.05).

**Table 2:** Laboratory findings in survivors and nonsurvivors (mean ± SD).

	Survivors *n* = 294	Nonsurvivors *n* = 28	*P* value
HCO_3_* (mmol/L)	27.08 ± 15.23	23.15 ± 8.42	N/S
pH∗	7.31 ± 0.23	7.27 ± 0.16	N/S
PCO_2_* (mm Hg)	52.06 ± 27.17	50.40 ± 23.04	N/S
Serum sodium (mEq/L)	141.7 ± 4.9	140.5 ± 8.7	N/S
Serum potassium (mEq/L)	4.34 ± 0.57	4.38 ± 0.71	N/S
Blood sugar (mg/dL)	121.0 ± 52.8	137.2 ± 72.6	N/S
Serum calcium (mEq/L)	8.50 ± 1.14	8.35 ± 0.59	N/S
Serum phosphorous	3.55 ± 1.34	2.86 ± 0.73	N/S
CPK (U/L)	1691 ± 3457	16058 ± 31749	N/S
LDH (U/L)	586.3 ± 746.0	5177 ± 4358	0.004
BUN (mg/dL)	25.7 ± 25.2	92.5 ± 101.0	0.01
Creatinine (mg/dL)	1.09 ± 1.11	3.19 ± 3.13	0.002
AST (IU/L)	150.4 ± 298.5	2789.5 ± 3761.4	0.04
ALT (IU/L)	162.6 ± 331.9	1881.7 ± 2209.9	0.03
WBC × 10^3^/*μ*L	11.2 ± 94.6	14.4 ± 52.3	N/S
Platelet × 10^3^/*μ*L	231 ± 93.35	173.08 ± 115.01	0.03

∗Based on arterial blood gas.

N/S: statistically nonsignificant (*P* > 0.05).

**Table 3:** ECG findings in studied cases (*n* = 151).

	Survivors *n* = 134	Nonsurvivors *n* = 16	*P* value
Prolonged P-R interval (PR ≥ 200 ms)	17 (12.7%)	2 (11.8%)	N/S
QRS prolongation (QRS ≥ 120 ms)	8 (6%)	3 (17.6%)	N/S
Prolonged Q-Tc interval (QTc ≥ 450 ms)	58 (43.3%)	10 (58.8%)	N/S
Left bundle branch block	0	1 (5.9%)	N/S
Right bundle branch block	15 (11.2%)	3 (17.6%)	N/S
Atrial fibrillation	0	2 (11.8%)	0.01
U wave	19 (14.2%)	1 (5.9%)	N/S
Sinus tachycardia	20 (14.9%)	6 (35.3%)	N/S
Premature atrial contraction	1 (0.7%)	0	N/S
Premature ventricular contraction	1 (0.7%)	0	N/S
ST depression	1 (0.7%)	0	N/S
Inversion of T wave	9 (6.7%)	0	N/S

N/S: statistically nonsignificant (*P* > 0.05).

**Table 4:** The relative frequency of complications.

	Total *n* = 322	Survivors *n* = 294	Nonsurvivors *n* = 28	*P* value
Disseminated intravascular coagulation	4 (1.2%)	1 (0.3%)	3 (10.7%)	0.002
Hepatic failure	5 (1.5%)	1 (0.3%)	4 (14.3%)	<0.001
Acute renal failure	16 (4.9%)	3 (1%)	13 (46.4%)	<0.001
Rhabdomyolysis	15 (4.6%)	7 (2.4%)	8 (28.6%)	<0.001
Pneumonia	15 (4.6%)	10 (3.4%)	5 (17.9%)	0.008
ALI/ARDS	5 (1.5%)	1 (0.3%)	4 (14.3%)	<0.001
Sepsis	2 (0.6%)	0	2 (7.1%)	0.007

ALI: acute lung injury.
